# Analysis of the Body Mass Index of Latino Patients With Bardet-Biedl Syndrome

**DOI:** 10.7759/cureus.86744

**Published:** 2025-06-25

**Authors:** Ricardo A Murati Calderon, Sebastián J Vázquez-Folch, Natalio Izquierdo

**Affiliations:** 1 Department of Ophthalmology, University of Puerto Rico, Medical Sciences Campus, San Juan, PRI; 2 School of Medicine, Universidad Central del Caribe, Bayamón, PRI; 3 Department of Surgery, School of Medicine, University of Puerto Rico, Medical Sciences Campus, San Juan, PRI

**Keywords:** autosomal recessive disorders, bardet-biedl syndrome, body mass index (bmi), genotype-phenotype association, obesity and overweight

## Abstract

Introduction: Bardet-Biedl syndrome (BBS) is a rare, autosomal recessive disorder characterized by multi-systemic manifestations, including truncal obesity, rod-cone dystrophy, polydactyly, and learning disabilities. The syndrome is associated with mutations in at least 28 genes, with *BBS1* being the most common. This study investigates the correlation between body mass index (BMI) and specific genetic mutations in a cohort of 27 Latino patients with BBS.

Methods: A chart review of 27 patients with BBS was conducted. Comprehensive ophthalmic evaluations, including best corrected visual acuity (BCVA), optical coherence tomography, and electroretinography, were performed. Genetic screening was conducted using next-generation sequencing with a focus on *BBS1*, *BBS7*, and *BBS19* mutations. BMI was classified into normal, overweight, and obese categories. Statistical analyses (chi-square and Kruskal-Wallis test) were used to evaluate the data.

Results: The cohort had a mean age of 39 years, with 21 patients (77.8%) carrying mutations in the *BBS1 *gene. Among the cohort, a total of 14 patients (51.9%) were classified as obese, 10 (37.0%) as overweight, and three (11.1%) as normal weight. Obesity was most common in patients with *BBS1* mutations, with 11 obese patients (78.6%) carrying *BBS1* mutations, while patients with *BBS7* and *BBS19* mutations exhibited lower rates of obesity. Among obese patients, 10 (71.4%) were homozygous for mutations in the associated genes, and three (21.4%) were compound heterozygotes. Gender did not show statistically significant differences in obesity prevalence; however, male patients were more frequently overweight.

Conclusion: This study confirms a strong association between *BBS1* mutations and obesity in BBS patients, with homozygous and compound heterozygous mutations contributing to more severe clinical manifestations. Further research is required to better understand the genotype-phenotype relationship and to explore the role of environmental factors, such as physical activity, in modifying obesity outcomes. Larger, more diverse studies are needed to validate these findings and to develop targeted therapeutic strategies for BBS-related obesity.

## Introduction

Patients with Bardet-Biedl syndrome (BBS) have multi-systemic clinical manifestations [1]. Primary diagnostic criteria include truncal obesity, rod-cone dystrophy (retinitis pigmentosa), post-axial polydactyly, genitourinary anomalies, learning disabilities, hypogonadism, and hypogonadotropism [1]. Minor clinical findings may also include speech and developmental delays, ataxia, diabetes mellitus, dental anomalies, liver and renal abnormalities, as well as anosmia or hyposmia [2].

Patients with the syndrome develop truncal obesity during the first decade of life due to hyperphagia [3]. The insatiable hunger in patients with the syndrome results from a faulty pathway involving the melanocortin 4 receptor (MC4R) in the hypothalamus [4]. Hyperphagia may contribute to behavioral problems in affected patients.

BBS is a rare heterogeneous group of inherited disorders affecting approximately one in 120,000 and one in 160,000 individuals in North America and Europe [5,6]. The syndrome is inherited in an autosomal recessive pattern and is associated with over 28 genes [2]. These include *BBS1*, *BBS2*, *BBS4*, *BBS5*, *BBS7*, *BBS9*, *BBS10*, *BBS12*, *BBS17*, *BBS18*, *BBS19*, and others involved in the formation and function of the primary cilium. Mutations in these genes associated with the syndrome lead to ciliopathy in the various affected tissues, contributing to diverse clinical manifestations of the syndrome [5].

The primary objective of this study is to analyze the distribution of BMI in patients with BBS and evaluate its association with specific genetic mutations in a Latino population. Given the underrepresentation of Latino individuals in BBS literature, this study aims to address potential ethnic variability in gene mutation prevalence and expression. BMI was selected as the clinical marker given its central role in the syndrome’s morbidity profile.

Considering previous reports linking *BBS1* to more pronounced metabolic dysfunction, we hypothesize that obesity will be most prevalent among patients with *BBS1* mutations, particularly those who are homozygous. By exploring this relationship, we report on the BMI of patients with various gene mutations leading to BBS in a Latino population.

## Materials and methods

This retrospective case series was conducted over a five-month period, from May to September 2024, at one major ophthalmology clinic in Puerto Rico: the San Francisco Ophthalmology Group in San Juan. We conducted a chart review of the medical records of patients diagnosed with the syndrome, examined by at least one of the authors (NJI), a trained ophthalmologist. All BBS patients underwent next-generation sequencing screening using a genotyping microarray provided by Invitae Corporation (San Francisco, CA), a College of American Pathologists-accredited and Clinical Laboratory Improvement Amendments-certified clinical diagnostic laboratory. Saliva kits with samples were submitted for processing by Invitae’s own inherited retinal diseases panel (Invitae Corporation). In this process, genomic DNA obtained from the submitted saliva sample is enriched for targeted regions using a hybridization-based protocol and sequenced using Illumina technology (Illumina, Inc., San Diego, CA). The Invitae report classifies variants as benign, pathogenic, or of unknown significance. Variant classification is based on population data, functional studies, computational predictions, clinical data, family segregation studies, evolutionary conservation, and evidence from databases such as ClinVar and in silico tools.

The inclusion criteria for this study were both a clinical diagnosis of BBS based on characteristic phenotypic features such as truncal obesity, retinal dystrophy, polydactyly, renal anomalies, or hypogonadism, and confirmation of pathogenic mutations in one or more BBS-associated genes identified through next-generation sequencing. Exclusion criteria included incomplete or insufficient medical documentation, lack of available or completed genetic testing, identification of only benign variants or variants of unknown significance without supporting pathogenic evidence, and mutations in genes unrelated to BBS. All patient data were anonymized to ensure confidentiality in compliance with ethical research standards.

A total of 27 patients with a confirmed clinical diagnosis of BBS and pathogenic variants in a BBS-associated gene were included in this study. Data were retrospectively collected from medical records and included demographic information (age and gender), past ocular and medical history, clinical features of BBS, best corrected visual acuity (BCVA), and results from genetic testing. Patients’ height and weight were recorded as documented in their medical charts, and BMI was calculated based on the most recent visit available in the record. While age and gender were incorporated into subgroup analyses, comorbidities were not included in the current analysis.

All patients underwent a comprehensive ophthalmologic examination using standardized protocols to ensure consistency across patient evaluations performed by at least one of the authors (NJI). BCVA was measured using a Snellen chart at 20 feet. Anterior and posterior segment examinations were conducted using a slit-lamp biomicroscope and an indirect ophthalmoscope. Macular optical coherence tomography was performed using the Heidelberg Spectralis system (Heidelberg Engineering, Heidelberg, Germany), and visual field testing was conducted using the Humphrey Field Analyzer (Carl Zeiss Meditec, Dublin, CA). Full-field electroretinography was carried out according to the International Society for Clinical Electrophysiology of Vision (ISCEV) standards. All patients demonstrated findings consistent with retinitis pigmentosa as part of their syndromic presentation. A descriptive analysis was conducted to report the frequency of identified BBS gene variants. Variant classification was confirmed using the ClinVar database. Statistical analysis included chi-square testing for categorical variables and the Kruskal-Wallis test for continuous variables. Missing data, when present, were handled through casewise deletion to preserve the integrity of statistical comparisons. A significance level of p < 0.05 was considered statistically significant.

Following the ethical principles governing medical research, the Institutional Review Board of the Universidad Central del Caribe, School of Medicine in Puerto Rico, conducted a thorough review of the research protocol. The protocol received approval (protocol number: 2025-5), confirming adherence to ethical standards and patient confidentiality in this retrospective study.

## Results

A total of 27 patients with the syndrome were evaluated, with ages ranging from 12 to 62 years (median = 39 years). There were 17 male and 10 female patients.

Twenty-one out of the 27 patients (77.8%) included in the study had mutations in the *BBS1* gene, four out of the 27 patients (14.8%) had mutations in the *BBS7* gene, and only two out of the 27 patients (7.4%) had mutations in the *BBS19* gene.

The patients’ weight ranged from 101 to 312 pounds (mean = 192 lbs.), and their height ranged from 57 to 76 inches (mean = 65 inches). Patients’ BMI was calculated. For the study, patients were classified based on their BMI: a BMI between 18.5 and 24.9 was normal, a BMI between 25 and 29.99 was overweight, and a BMI of 30 or higher was obese.

Table 1 summarizes the BMI distribution of patients with the syndrome, including all types. Three patients (11.1%) were classified as having a normal weight, 10 patients (37.0%) were classified as overweight, and 14 patients (51.9%) were classified as obese. Hence, most patients within our cohort were classified as obese.

**Table 1:** BMI classification among patients with BBS. BMI: body mass index; BBS: Bardet-Biedl syndrome.

BMI category	Frequency	Percentage (%)
≤25 (normal)	3	11.1
25-29.99 (overweight)	10	37.0
≥30 (obese)	14	51.9
Total	27	100.0

Table 2 summarizes BMI categories according to the genetic subtype in patients with the syndrome. Among the three patients with normal weight, one (33.3%) had mutations in the *BBS1*, *BBS7*, and *BBS19* genes, respectively. Regarding the overweight category, nine patients (90%) had *BBS1* gene mutations, while only one (10%) had *BBS7* gene mutations; none of the patients with the *BBS19* mutation were classified as overweight. In the obese category, most patients (11, 78.6%) had the *BBS1* gene mutation, followed by two patients (14.3%) with mutations in *BBS7* and only one patient (7.1%) with a mutation in *BBS19*. However, there was no statistically significant difference between these categories.

**Table 2:** Distribution of BMI categories by BBS gene mutation variant. % within BMI: Percentage of individuals within each BMI category who carry the specified gene mutation. % within gene: Percentage of individuals with a specific BBS gene mutation who fall into a given BMI category. Each cell presents the number of patients with a specific BBS gene mutation, followed by the percentage within the BMI category and within the gene mutation group, respectively. BMI: body mass index; BBS: Bardet-Biedl syndrome.

BMI category	BBS1 count (% within BMI, % within gene)	BBS7 count (% within BMI, % within gene)	BBS19 count (% within BMI, % within gene)	Total
≤25 (normal)	1 (33.3%, 4.8%)	1 (33.3%, 25.0%)	1 (33.3%, 50.0%)	3
25–29.99 (overweight)	9 (90.0%, 42.9%)	1 (10.0%, 25.0%)	0 (0.0%, 0.0%)	10
≥30 (obese)	11 (78.6%, 52.4%)	2 (14.3%, 50.0%)	1 (7.1%, 50.0%)	14
Total	21 (77.8%, 100.0%)	4 (14.8%, 100.0%)	2 (7.4%, 100.0%)	27

Table 3 compares the BMI of homozygous, heterozygous, and compound heterozygous patients for mutations leading to BBS. All patients with normal BMI (≤25) were homozygotes. Among the overweight patients (BMI: 25-29.99), three (30.0%) were homozygous, and seven (70.0%) were compound heterozygous, with no overweight heterozygotes. In the obese category (BMI ≥ 30), 10 (71.4%) patients were homozygous (p = 0.074), one (7.1%) patient was heterozygous, and three (21.4%) patients were compound heterozygotes.

**Table 3:** Comparison of BMI categories across homozygous, heterozygous, and compound heterozygous BBS mutations. Homozygous: Individuals with two identical mutations. Heterozygous: One mutant allele and one normal allele. Compound heterozygous: Two different mutant alleles at the same locus. BMI: body mass index; BBS: Bardet-Biedl syndrome.

BMI category	Homozygous count (% BMI)	Heterozygous count (% BMI)	Compound heterozygous count (% BMI)
≤25 (normal)	3 (100.0%)	0 (0.0%)	0 (0.0%)
25-29.99 (overweight)	3 (30.0%)	0 (0.0%)	7 (70.0%)
≥30 (obese)	10 (71.4%)	1 (7.1%)	3 (21.4%)
Total	16 (59.3%)	1 (3.7%)	10 (37.0%)

Table 4 presents the distribution of BMI categories by gender in patients with BBS. All patients with normal BMI (≤25 kg/m²) were male. In the overweight group (25-29.99 kg/m²), seven (70.0%) were male and three (30.0%) were female. In the obese category (≥30 kg/m²), the distribution was equal between males and females (50.0% each). While male patients were more frequently overweight than females, this difference did not reach statistical significance (p = 0.093).

**Table 4:** Distribution of BMI categories by gender in patients with BBS. BMI: body mass index; BBS: Bardet-Biedl syndrome; % BMI: proportion of individuals within each BMI category by gender.

BMI category	Female count (% BMI)	Male count (% BMI)	Total
≤25 kg/m^2^ (normal)	0 (0.0%)	3 (100.0%)	3 (100.0%)
25-29.99 (overweight)	3 (30.0%)	7 (70.0%)	10 (100.0%)
≥30 (obese)	7 (50.0%)	7 (50.0%)	14 (100.0%)
Total	10 (37.0%)	17 (63.0%)	10 (37.0%)

## Discussion

Studies by Mujahid and co-workers have demonstrated that mutations in the *BBS1* gene are associated with the most severe clinical manifestations of metabolic dysfunction in BBS patients, including obesity, insulin resistance, and dyslipidemia [7]. In our study, a high frequency of obesity was found among patients with the syndrome, as well as with the specific* BBS1 *​​​​​​mutation. Most of our cohort was classified as obese, which aligns with the well-established association between BBS and obesity, further supporting the connection between the syndrome and obesity [8].

Previous studies have reported a clear correlation between obesity and mutations in the *BBS1* gene in North American and European populations [7]. Consistent with these findings, our study also found that most obese patients carried mutations in the *BBS1* gene, as shown in Table 2. In contrast, patients with mutations in the *BBS7* and *BBS19* genes had a significantly smaller proportion of obesity. These findings further support the association between *BBS1* mutations and a higher prevalence of obesity in patients with the syndrome. Unfortunately, obesity in these patients is strongly associated with additional complications such as insulin resistance, diabetes, and renal disease [7,9].

Nawaz and colleagues described obesity in homozygous carriers of mutations in genes associated with BBS, correlating allelic mutations and the severity of clinical manifestations [10]. In our study, 10 (71.4%) of all obese patients were homozygous for mutations in BBS-related genes, such as *BBS1*, *BBS7*, and *BBS19 *(95% CI: 48%-95%). Among homozygous, 10 patients (62.5%) were obese (95% CI: 39-86%). Additionally, three (21.4%) of all obese patients with the syndrome were compound heterozygous for these mutations, while only one (7.1%) of the obese patients with the syndrome was heterozygous. These findings suggest that homozygosity for mutations in genes related to the syndrome may be associated with a more severe clinical phenotype, including metabolic dysregulation and a higher risk of obesity. Perea-Romero and coworkers recently demonstrated that BBS patients with a higher mutational load, including biallelic and triallelic combinations, exhibited more severe phenotypic features, supporting the relevance of exploring allelic complexity in understanding clinical variability [11]. To our knowledge, this is the first study to report the association between homozygosity and compound heterozygosity with obesity in Latino patients with BBS.

Pomeroy and co-workers previously reported that obesity occurred more frequently in female patients with BBS [8]. In our study, obesity occurred at similar rates in both male and female patients with the syndrome. This discrepancy may reflect differences in physical activity levels between male and female patients with BBS, which could influence the degree of obesity observed. Further research is needed to investigate the potential gender-based differences in physical activity levels and their impact on metabolic outcomes in BBS [12].

Several mechanisms may contribute to the high prevalence of obesity in patients with BBS. The specific role of *BBS1* in leptin signaling, which typically regulates appetite control and fat storage, may account for the observed higher rates of obesity in these patients [4]. Recent studies by Blaess and co-workers have shown that the MC4R pathway is disrupted in patients with the syndrome, leading to hyperphagia and, thus, obesity [13]. Furthermore, the sedentary lifestyle associated with visual impairment in BBS patients may exacerbate weight gain, further contributing to metabolic imbalances [2,4,13,14].

The study's limitations include a small sample size. This is due to the syndrome being a rare disease. Further studies with larger, more diverse populations are needed to validate these findings. The small number of patients presenting with mutations in the *BBS7* and *BBS19* genes limits conclusions regarding their association with obesity. The underrepresentation of *BBS7* and *BBS19* mutations in our Latino sample may reflect underlying ethnic variability in BBS gene distribution, which has been described in prior genetic studies involving BBS [15,16]. However, this observation should be interpreted cautiously, as our study lacks robust comparative genetic data from other populations. Further population-based studies are needed to confirm whether this pattern is consistent across broader and more diverse ethnic groups. Another key limitation of the study is its retrospective design, which may introduce selection bias. Because the data were derived from medical records, we were unable to systematically collect or control for potential confounding variables such as dietary intake, physical activity, and socioeconomic status, all of which can influence BMI.

Further studies are warranted to confirm whether this effect is consistent across large and more diverse populations. Subsequently, more extensive, diverse studies are essential to better understand the genotype-phenotype correlation and its implications for clinical practice. Genetic screening for BBS mutations may help individualize treatment approaches early in the disease course, especially for managing obesity and other metabolic disorders in those with the BBS1 mutation. Recognizing patients at higher risk for early-onset obesity based on their genetic profile could facilitate early interventions, including dietary management, lifestyle modifications, and metabolic therapies. In the future, longitudinal studies assessing metabolic interventions tailored to specific BBS genotypes could provide guidance on effective therapeutic strategies.

## Conclusions

This study offers one of the first genotype-phenotype analyses of BBS in a Latino population, with results showing that *BBS1* was the most prevalent mutated gene, particularly among obese patients. Notably, the strongest association with obesity was observed in patients who were homozygous or compound heterozygous for *BBS1* mutations. These findings support previous literature while introducing novel insight into the potential severity of metabolic manifestations based on zygosity. Additionally, our findings suggest that obesity occurred equally in both male and female patients, contrasting with previous studies that reported a higher prevalence in females, potentially indicating the influence of factors like physical activity levels.

Furthermore, the relative underrepresentation of *BBS7* and *BBS19* mutations in our cohort raises questions about ethnic variability in gene distribution, though further comparative studies are needed to confirm this. By stratifying phenotypic expression by genotype and highlighting the burden of obesity among genetically confirmed cases, this study contributes meaningful data to the limited literature on BBS in underrepresented populations. Future research should aim to include larger, longitudinal, and ethnically diverse cohorts to further explore the metabolic and genetic landscape of BBS. Understanding the full genetic spectrum of obesity in BBS is crucial for developing targeted therapeutic strategies for this multi-systemic disorder.
